# Mental health of Covid-19 risk groups during the first Covid-19 lockdown in Germany: a cross-sectional study

**DOI:** 10.1186/s12889-022-13593-z

**Published:** 2022-06-14

**Authors:** Daniel Deimel, Thorsten Köhler, Janina Dyba, Niels Graf, Christine Firk

**Affiliations:** 1 German Institute for Addiction and Prevention Research, Catholic University of Applied Sciences of North Rhine–Westphalia, Konrad-Adenauer-Ufer 79-81, 50668 Cologne, Germany; 2 Department of Social Work, Catholic University of Applied Sciences of North Rhine–Westphalia, Robert-Schuman-Str. 25, 52066 Aachen, Germany; 3 Department of Addictive Behavior and Addiction Medicine, Medical Faculty, LVR-Hospital Essen, University of Duisburg-Essen, Essen, Germany; 4 School of Public Health, Bielefeld University, Universitätsstr. 25, 33615 Bielefeld, Germany

**Keywords:** Covid-19, Pandemic, Mental health, Covid-19 risk group, Germany

## Abstract

**Background:**

The ongoing Covid-19 pandemic not only threatens physical health, but also affects the mental health of people. Yet, health consequences of the pandemic do not affect all members of society equally. We therefore assessed the mental health burden of individuals who are at increased risk of severe illness from Covid-19 compared to individuals who are at low risk of severe illness during the first lockdown (March, 2020) in Germany. Furthermore, we investigated variables mediating the effect of being an individual at increased risk of serve illness on depression.

**Methods:**

Adult German residents (*n* = 2.369) provided responses to a cross-sectional online survey about risk factors for of severe illness from Covid-19 and various aspects of mental health during the first lockdown in Germany. For data collection, standardized and validated self-report measures were used and for data analysis Mann-Whitney U-tests as well as regression and mediation analyses were performed.

**Results:**

The results clearly show that the mental health burden is higher among individuals at increased risk of severe illness from Covid-19 compared to individuals at low risk of severe illness from Covid-19. Moreover, our findings indicate that the association between Covid-19 risk status and depressive symptoms is mediated by concerns about mental health, anxiety and loneliness in a causal effect chain.

**Conclusions:**

Individuals at increased risk of severe illness from Covid-19 have an increased need for psychosocial support during times of lockdown. Future public health policies should pay special attention to these individuals and support them by targeted offers. More research, however, is needed on possible long-term consequences of social distancing on mental health.

## Introduction

The most recently discovered coronavirus, known as severe acute respiratory syndrome coronavirus 2 (SARS-CoV-2), has spread globally within a few months after its first identification in December 2019 [1]. The World Health Organization (WHO) declared the Covid-19 disease caused by the virus as a pandemic on March 11th 2020. In Germany, the first case of Covid-19 was confirmed on January 27th 2020 [2]. First infection clusters emerged in the federal states North Rhine-Westphalia and Bavaria throughout February 2020 [3]. Subsequently, Covid-19 cases increased rapidly, culminating in about 6016 new cases on March 16th 2020 [4]. As of June 2020, by the end of the so-called “first wave”, 183,594 persons had been diagnosed with a SARS-CoV-2 infection in Germany and the number of deaths registered in this group amounted to 8555 [5]. The cumulative rate of officially recognized Covid-19-associated hospitalizations in Germany is 10% [6].

**Fig. 1:**
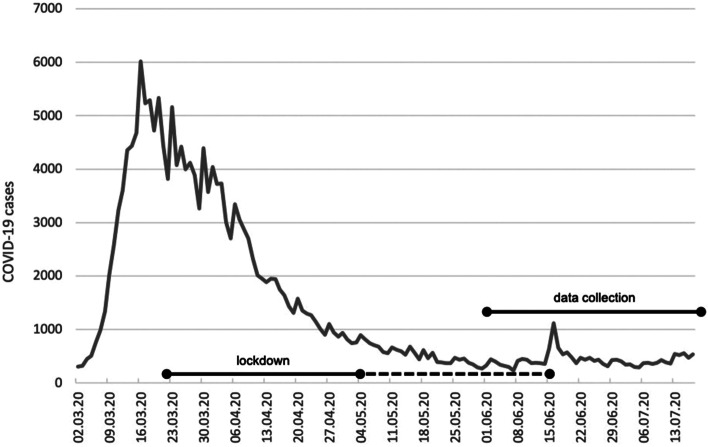
First Covid-19 lockdown in Germany. Sources: Own elaboration based on data from RKI [4]

Older people above the age of 50-60 and people with underlying medical conditions, such as heart conditions, chronic obstructive pulmonary disease (COPD), or obesity are at increased risk of severe illness from Covid-19 [7, 8]. On March 22nd 2020, the German government imposed a first lockdown to reduce infection rates and thus protect these vulnerable groups and maintain the proper functioning of the health care system. This lockdown included the closing of schools, stores, restaurants, bars, clubs, social venues and prohibited any form of mass gatherings. In addition, citizens were urged to minimized personal social contact and keep a minimum distance of 1.5 m from one another [9]. It lasted until May 4th 2020 and associated regulations were gradually eased by June 15th 2020 (see Fig. 1).

These governmental actions aim at a reduction of social contacts. Social distancing however may be associated with a substantial mental health burden and there is evidence for an association between social isolation and (mental) health problems [10, 11]. This is also supported by recent studies showing that the Covid-19 pandemic and related regulations are associated with increases in anxiety, depression and psychological distress [12–14]. The increase in mental health problems may in turn also favor dysfunctional coping and emotion regulation strategies such as substance use [15]. Even though these mental health impacts of the Covid-19 pandemic may be more significant for those who are prone to psychological problems [16], previous studies have not taken into account the mental health of individuals at increased risk of severe illness from Covid-19 due to their age or underlying medical conditions [7, 8]. Individuals at risk of severe illness from Covid-19 may be more worried about their own health and therefore avoid social contacts to reduce the risk of a Covid-19 infection. Previous studies demonstrated a relationship between concern of COVID pandemic and feelings of loneliness [17, 18]. This may increase feelings of loneliness, which in turn may result in mental health problems such as depression [19].

Hence, the primary aim of the present study is the investigation of the mental health burden of individuals who are at increased risk of severe illness from Covid-19 (high risk group for Covid-19, HRGC) compared to individuals who are at low risk of severe illness (low risk group for Covid-19, LRGC). The central hypothesis is that individuals of the HRGC are more anxious and experience more depressive symptoms due to the pandemic than individuals of the LRGC. Based on associations between anxiety, depression, and substance use, we moreover expect that HRCG individuals report enhanced substance use. Furthermore, the second aim of the current study is to investigate whether the hypothesized increase in depressive symptoms in the HRGC group is mediated by concerns about own mental health, anxiety, stress and loneliness.

## Methods

### Study design and data collection

Cross-sectional data were collected via an online survey from June 1st 2020 until July 17th 2020. The survey was developed in LimeSurvey (LimeSurvey GmbH, Hamburg). The weblink of the survey was included in an advert that was promoted on the websites and social media platforms of several German social service organisations and associations (German AIDS Service Organisations, German Society for Social Psychiatry, German Federation of Telephone Emergency Services, German Federation for Social Work in the Healthcare System, German Society for Social Work in Addiction Aid).

To be able to participate in the study, participants had to be at least 18 years and have sufficient knowledge of the German language. Participants did not get any compensation for participating in the survey. In total, 3154 people were reached through the online survey. For this study, a subset of participants (*n* = 2.369) has been analysed for the comparison of the mental health burden of HRGC and LRGC participants.

### Measures

The survey started with comprehensive participant information and consent forms. This introductory part was followed by 132 items on sociodemographic variables, participants’ mental health status, their perceptions of the Covid-19 pandemic and the governmental actions designed to encounter the pandemic.

#### Mental health

All items on mental health were part of standardized and validated self-report measures. Subscales of the German version of the Patient Health Questionnaire (PHQ-D) [20] were used to assess levels of depression (PHQ-9) (Kromke et al. 2006), anxiety (General Anxiety Disorder-7, GAD-7) [21] and somatisation (Patient Health Questionnaire-15, PHQ-15) [22]. The PHQ-9 scale assesses severity of depressive symptoms with a maximum score of 27. GAD-7 measures symptoms of anxiety with a maximum of 21. A score of 10 or above on each of the two scales points to an at least moderate major depressive episode and moderate levels of clinical anxiety [21, 23]. The items of the PHQ-15 scale include the most prevalent DSM-IV somatization disorder somatic symptoms. The total PHQ-15 scale has a maximum score of 30 and a score of 10 and above represent a moderate level of somatization [22]. The internal reliability of the PHQ-9 was with a Cronbach’s α of 0.90 similar to other studies (0.86-0.89) [23]. The internal consistency of the GAD-7 was with a Cronbach’s α = 0.91 similar to another study (0.89) [21] and of PHQ-15 with a Cronbach’s α = 0.81 equal to another study (0.82) [22].

#### Suicidality

Suicidality was assessed by the first item of the German version of the Suicide Behaviours Questionnaire – Revised (SBQ-R) which is acknowledged as a reliable instrument to measure suicidal risk (“Have you ever thought about or tried to take your own life?” = never (1); I had only a fleeting thought about it (2); I had at least 1 intention to kill myself, but I did not try (3); I had at least 1 intention to kill myself and I really wanted to die (3); I tried to kill myself, but I did not want to die (4); I tried to kill myself, and I really wanted to die (5)). A score of 3 and higher represents an increased risk of suicide [24, 25]. This item was complemented by a question on suicidal ideation during the first lockdown in Germany (“How often have you thought about killing yourself during the lockdown?”).

#### Loneliness

Emotional and social loneliness were surveyed by the 11-item De Jong Gierveld Loneliness Scale with a maximum score ranges from 0 to 22 [26]. The internal consistency of the Loneliness-Scale was with a Cronbach’s α = 0.77.

#### Social support

The level of social support was assessed with the help of the Oslo 3 Social Support Scale (OSSS-3). The score ranges from 3 to 14. A score of 12 and above represent a strong social support. The internal consistency of the OSSS-3 was with a Cronbach’s α = 0.66 simliar to another study (0.64) [27, 28].

#### Drug use

Moreover, the use of alcohol, nicotine and a range of illegal substances during the last 12 months as well as changes in substance use during the lockdown were assessed by asking the participants which substances they used in the last 12 months, respectively during the first lockdown.

To differentiate between HRGC and LRGC participants, risk factors for an increased risk of severe illness from Covid-19 were assessed by the criteria of the Robert Koch Institute [29] which include smoking, obesity, cardiovascular diseases, chronic lung diseases, diabetes mellitus, cancer, and a compromised immune system. If at least one of these criteria was met, participants were included in the HRGC group.

### Statistical analysis

We used a subset of the dataset and included all participants who gave information about their Covid-19 risk profile (*n* = 2.369). The analyses presented here compare two groups: (i) individuals at increased risk of severe illness from Covid-19 (*n* = 1.136; HRGC group) and (ii) Individuals at low risk of severe illness from Covid-19 (*n* = 1.233; LRGC group). Data analysis was conducted using IBM SPSS Statistics 25.0 (IBM corp., Armonk, USA). Significance level of *p* < 0.05 was considered in all analyses.

For group comparisons Mann-Whitney U-tests were performed for ordinal and non-normally distributed data. Cohen’s d is reported as the estimated effect size for statistically significant results. The distribution of categorical variables was assessed by Chi-square tests. Spearman’s correlation coefficients were used to determine correlations between ordinal variables and non-normally distributed continuous variables. Pearson’s correlation was used for normally distributed continuous variables. Linear regression analysis was used to explore predictors for depressive symptoms. Additionally, mediation analysis using PROCESS macro [30] for SPSS 25 (IBM corp., Armonk, USA) was run to explore whether concerns about one’s own health, anxiety and feelings of loneliness mediated depressive symptoms. Multiple mediator models were performed to estimate indirect effects [31]. All analyses were based on 5000 bootstrapped samples. An indirect effect was considered significant when the 95% bias-corrected confidence interval did not include zero [30].

## Results

### Sample characteristics

Of the 3154 persons who commenced the survey, 2.369 participants completed questions on Covid-19 risk factors (75.11%). 47% (*n* = 1291) of those participants were classified into the HRGC. Data of non-completers were included on a pairwise basis, resulting in a different number of responses per analysis (for details on the sociodemographic characteristics of the HRGC and the LRGC, see Table 1).

**Table 1:** Sociodemographic characteristics

Variable	COVID-19 risk group		Non-COVID-19 risk group		***p-value***
	***N***	***M*** **(*SD*)**	***N***	***M*** **(*SD*)**	***t-test***
Age	1136	46.1 (14.8)	1233	39.4 (14.6)	.460
	***N***	**%**	***N***	**%**	**X**^**2**^
Gender	1137		1236		< .001
Female	706	62.1	896	72.5	
Male	412	36.2	323	26.1	
Diverse	19	1.7	17	1.4	
Employment status	1291		1.406		< .001
Full-time employed	483	37.4	501	35.6	
Part-time employed	298	23.1	386	27.5	
Retired	196	15.2	77	5.5	
Student	154	11.9	325	23.1	
Unemployed	73	5.7	28	2.0	
Other	87	6.7	89	6.3	
Monthly net income	1101		1188		< .001
< 1.000 Euros	248	22.5	357	30.1	
1.000-2.000 Euros	382	34.7	362	30.5	
2.000-3.000 Euros	289	26.2	311	26.2	
More than 3.000 Euros	182	16.5	158	13.3	
Education	1133		1237		.037
University or university of applied sciences diploma	557	49.2	677	54.7	
Completed vocational education	152	13.4	133	10.8	
Completion of secondary school	417	36.8	421	36.8	
Other/none	7	0.6	6	0.5	

### Mental health measures

In total, 30.9% of the participants of both groups reported symptoms of a moderate depression on the PHQ-9 scale (score of 10 or higher). The median PHQ-9 score was significantly higher in the HRGC than in the LRGC group. 35.6% of the HRGC participants and 26.6% of the LRGC participants had a PHQ-9 score of 10 or higher and, therefore, exhibited moderate depressive symptoms. Compared to the LRGC, the median GAD-7 score of the HRGC was also significantly higher. Here, 29.6% of the HRGC participants and 21.4% of the LRGC participants showed at least moderate levels of generalized anxiety disorders (GAD-7 score ≥ 10). A similar pattern applies to somatic symptoms. The median PHQ-15 score was again significantly higher in the HRGC than in the LRGC group. 15,6% of the HRGC participants and 7.6% of the LRGC participants exhibited at least moderate somatic symptoms (PHQ-15 score ≥ 10). In total, 14.4% of the participants showed an elevated risk for suicide (SBQ-R Item 1 ≥ 3). Again, an elevated risk for suicide was significantly higher in the HRGC than in the LRGC (19.5% vs. 9.7%) group. The same results can be found for the median suicidal ideation during the lockdown (see Table 2).

**Table 2:** Mental health

Variable	HRGC			LRGC			Test statistic	Significance	Effect size
	***N***	**Mdn (IQR)**	**M**	***N***	**Mdn (IQR)**	**M**	**Mann-Whitney U**	***p-value***	***r***
Depression (PHQ-9 score)	1083	6.00 (9.00)	7.9	1182	5.00 (7.00)	6.39	552,002,5	< .001	0.12
Anxiety (GAD-7 score)	1078	6.00 (8.00)	7.18	1189	5.00 (7.00)	6.04	565,787,0	< .001	0.10
Somatization (PHQ-15 score)	846	4.00 (6.00)	5.11	1017	3.00 (4.00)	3.73	349,393,5	< .001	0.16
	***N***	**%**		***N***	**%**		**X**^**2**^	***p-value***	**Phi**
Depression (PHQ-9 score ≥ 10)	351	35.6		282	26.6		19,203	< .001	0.097
Anxiety (GAD-7 score ≥ 10)	319	29.6		255	21.4		19,838	< .001	0.094
Somatization (PHQ-15 score ≥ 10)	132	15.6		77	7.6		29,910	< .001	0.127
Suicidality lifetime SBQ-R Item 1 ≥ 3	227	19.5		124	9.7		47,544	< .001	0.435
	***N***	**Mdn (IQR)**	**M**	***N***	**Mdn (IQR)**	**M**	**Mann-Whitney U**	***p-value***	***r***
Suicidal thoughts during lockdown	535	1.0 (1.00)	1,76	432	1.0 (1.00)	1,59	107,184,5	.026^*^	0.125

### Substance use during lockdown

There were no significant differences between the HRGC and the LRGC group for alcohol use during the lockdown. In contrast, the use of nicotine and THC during the lockdown differed significantly between the two groups. 20.1% of the HRGC reported an increased use of nicotine during the lockdown compared to 6.1% of the LRGC participants. An increased use of THC during the lockdown was reported by 6.7% of the HRGC individuals compared to 2.1% of the LRGC participants (see Table 3).

**Table 3:** Descriptive statistics and X^2^ results for substance use in the HRGC and LRGC

Variable	HRGC		LRGC		***p-value***	Effect size
	***N***	%	***N***	%	***X***^**2**^	Phi
Substance use in the last 12 months						
Alcohol	1056	97.4	1151	93.4	.190	
Nicotine	618	62.5	302	29.3	< .001	0.333
THC	292	30.5	186	17.3	< .001	0.155
Cocaine	56	5.3	23	1.9	< .001	0.093
Amphetamines	69	6.5	29	2.4	< .001	0.101
Methamphetamines	23	2.1	8	0.7	.002	0.064
Ecstasy	65	6.2	33	2.7	< .001	0.084
Alcohol use during lockdown	1137		1259		.046	0.064
No use	231	20.3	226	18.0		
Less than before	187	16.4	210	16.7		
No change	416	36.6	500	39.7		
Slightly more than before	228	20.1	269	21.4		
Significantly more than before	75	6.6	54	4.3		
Nicotine use during lockdown	1106		1177		< .001	0.391
No use	529	47.8	988	83.9		
Less than before	69	6.2	44	3.7		
No change	286	25.9	100	8.5		
Slightly more than before	160	14.5	32	2.7		
Significantly more than before	62	5.6	13	1.1		
THC use during lockdown	1069		1185		< .001	0.148
No use	859	80.4	1064	89.8		
Less than before	39	3.6	28	2.4		
No change	100	9.4	68	5.7		
Slightly more than before	50	4.7	23	1.9		
Significantly more than before	21	2.0	2	0.2		

### Loneliness, social support and professional assistance

Loneliness was significantly higher in the HRGC group compared to the LRGC (7.3% vs. 3.8%). The level of perceived social support did not differ significantly between both groups (see Table 4).

**Table 4:** Dealing with the pandemic

Variable	HRGC			LRGC			Test statistic	Significance	Effect size
	***N***	**%**		***N***	**%**		***X***^**2**^	***p-value***	**Phi**
Loneliness (11-item De Jong Gierveld Loneliness Scale) score ≥ 16	77	7.3		45	3.8		13,005	< .001	0.076
Social support (OSSS-3) score ≥ 12	250	22.4		300	25.0		2260	.133	
	***N***	**Mdn (IQR)**	**M**	***N***	**Mdn (IQR)**	**M**	**Mann-Whitney-U**	***p-value***	***r***
Burdens of social distancing	1289	4.00 (3.00)	3.51	1408	3.00 (2.00)	3.44	881,196,0	.185	
Meaningfulness of social distancing	1284	5.00 (2.00)	4.88	1402	5.00 (2.00)	4.77	840,222,0	.002	0.060
Concerns about the pandemic...									
Concerns about own health	1282	3.00 (2.00)	2.94	1398	2.00 (2.00)	2.3	685,748,5	< .001	0.208
Concerns about the health of friends	1271	4.00 (2.00)	4.08	1402	4.00 (2.00)	3.96	844,683,0	.018	0.045
Concerns about own financial situation	1271	2.00 (2.00)	2.39	1406	2.00 (2.00)	2.14	827,655,5	< .001	0.067
Concerns about the German healthcare system	1279	3.00 (2.00)	2.84	1396	2.00 (3.00)	2.63	822,044,0	< .001	0.070
Concerns about the German economy	1277	4.00 (2.00)	3.99	1396	4.00 (2.00)	3.8	818,631,5	< .001	0.072
Concerns about the German political system	1274	4.00 (2.00)	3.96	1383	4.00 (2.00)	3.79	820,626,5	< .001	0.060

*p < .05

**p < .01

***p < .001

Feelings of stress associated with social distancing did not differ significantly between both groups. HRGC individuals, however, were significantly more likely to perceive government actions to encounter Covid-19 as legitimate and meaningful than LRGC participants. Generally, HRGC individuals were significantly more concerned about the pandemic than LRGC participants. Here, HRGC individuals were significantly more worried about their own health, the health of their friends, the health system in Germany, their financial situation as well as the German economic and political system than LRGC participants (see Table 4).

### Factors contributing to depressive symptoms during the lockdown

Bivariate correlations showed a significant positive association between depression, anxiety, loneliness and the perceived stress level due to social distancing (see Table 5).

**Table 5:** Bivariate correlations of loneliness, depression and stress due to social distancing

		1	2	3	4
1	Depression	1			
2	Anxiety	.824^**^	1		
3	Loneliness	.591^**^	.477^**^	1	
4	Stress due to social distancing during lockdown	.406^**^	.400^**^	.428^**^	1

** *p* < 0.01

Linear regression was used to identify predictors of depressive symptoms during the lockdown. Being male (*β = −.025, p =* .044), younger age (*β = −.041, p* = .001), being a HRGC individual (*β = .052, p <* .001), loneliness (*β = .238, p* < .001), lower worries about the own health (*β = −.030, p =* .020) as well as anxiety (*β = .681, p* < .001) were significantly associated with depressive symptoms during the lockdown. Perceived stress due to social distancing did not significantly predict depression (*β = .014, p* = 314). The overall regression was statistically significant (R^2^ = .732, *F*(7-1867) = 730,778, *p* < .001) (see Table 6).

**Table 6:** Serial logistic regression model for variables associated with depression (*n* = 1875)

Variable	Depression (PHQ-9 Score)			
	***β***	Standard error	***T*** Value	Significance
Gender, Male	−.025	.156	−2.011	.044
Age	−.041	.005	−3.233	.001
HRGC individual	.052	.150	4.103	<.001
Loneliness	.238	.021	15.735	<.001
Concerns about own health	−.030	.053	−2.335	.020
Anxiety (GAD-7 Score)	.681	.018	43.429	<.001
Stress due to social distancing during lockdown	.014	.057	1.007	.314

Mediation analysis using PROCESS macro for SPSS 25 (IBM corp., Armonk, USA) was run to explore variables mediating the effect of being a HRGC individual on depression. All mediation analyses were controlled for age and gender as covariates.

First, a parallel mediation model was run to test whether the effect of being a HRGC individual (X) on depression (Y) was mediated by concerns about own health (M_1_), by feelings of loneliness (M_2_), by stress due to social distancing (M_3_) or by anxiety (M_4_). The results of the mediation analysis (total effect: 2.02, 95% CI: 1.48-2.56; direct effect: .573, 95% CI: .278-.868) demonstrated that the indirect effects were only significant for concerns about own health (M_1_: CI:-.142--.009;) feelings of loneliness (M2: 95% CI:.195-.475;) and anxiety (M_4_: 95% CI: .815 -1.55), but not for stress due to social distancing (M_3_: 95% CI: −.012-.031).

**Fig. 2:**
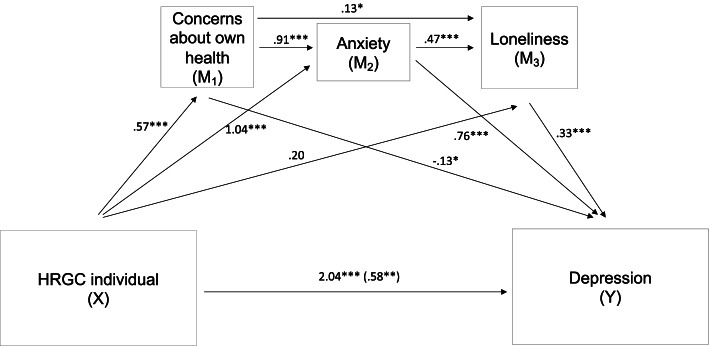
Serial multiple mediator model. *Notes:* Significant indirect effect of X on Y through M1, M2 and M3 in serial (total effect: 2.04, 95% CI: 1.49-2.57; direct effect: 95% CI: .289-.879; indirect effect: 95% CI: .055-.113). Unstandardized beta coefficients are presented. For the direct effect unstandardized coefficients (before and after the mediators (in parentheses) were added to the model) are presented. Mediation analyses was controlled for gender and age. ^*^p < .05^**^ *p* < .01, ^***^ *p* < .001

Based on this mediation model, a serial multiple mediation model was run. Here, mediators are linked together in a causal effect chain, with mediators allowing to influence each other (M_1_ (concerns about own health) → M_2_ (anxiety) → M_3_ (loneliness)). The mediation model showed that the association between Covid-19 risk group and depression was mediated by this serial mediation chain (total effect: 2.04, 95% CI: 1.49-2.57; direct effect: 95% CI: .289-.879; indirect effect: 95% CI: .055-.113) with concerns about own health being linked to anxiety and this in turn being associated with feelings of loneliness (see Fig. 2).

## Discussion

According to estimations of the RKI, 52% of all persons living in Germany aged 15 or older belong to a group at risk for severe illness from Covid-19 [32]. The proportion of individuals at increased risk for severe illness from Covid-19 (HRGC) in this study was 47% and thus remarkably higher. The primary aim of this study was to investigate differences in mental health problems (such as depression, anxiety, psychosomatic symptoms and substance use) during the Covid-19 pandemic in HRGC individuals compared to LRCG individuals. In addition, we discuss the relation of these findings in regard to the general German population. We found that 35.9% of the HRGC individuals reported moderate depressive symptoms compared to 26.6% of the LRGC individuals. The proportion of persons with at least moderate depressive symptoms in the HRGC group is remarkably higher than in the LRGC group and four times as high as in the German general population [33]. Regarding the overall rate of depression during the time of the first lockdown in Germany, rates were estimated to have increased to 14.3% (PHQ-2 score ≥ 3) in the general population [12]. Yet, more than twice as many individuals in the HRCG group reported depressive symptoms. Moreover, 29.6% of the HRGC individuals exhibited clinically relevant symptoms of a generalized anxiety disorder in the presented study, while this applies to only 21.4% of the LRGC group. Again, this rate is considerably higher than in the general German population, where the prevalence is estimated at 5,9% [34]. Several studies confirm an increase of generalized anxiety disorders during the first period of the pandemic. A German study [12] reported at least moderate symptoms of generalized anxiety disorders (GAD-7 score ≥ 10) in 16.8% of the participants, which is still a substantially lower rate than in our HRGC group. In terms of somatic symptoms 15.6% of the HRGC individuals and 7.6% of the LRGC individuals showed clinically relevant somatic symptoms in this study, compared to only 9.3% in the German general population [22]. In addition, 19.5% of the HRGC individuals and 9.7% of the LRGC individuals reported an elevated risk for suicide. Hence, the proportion of individuals with and increased risk for suicide is three times higher in the HRGC group than in the German general population [24].

Based on previous studies [19, 35] pointing to the importance of feelings of loneliness for depression, the second aim of the current study was to investigate the association of concerns about own health, anxiety, perceived loneliness, and stress due to lockdown measures with depressive symptoms. Using mediator models, we demonstrated that the direct effect of being an HRGC individual on depression was mediated by concerns about own health, anxiety and feelings of loneliness. In a serial mediation model, an indirect causal effect chain was observed showing that being an HRCG individual was related to concerns about own health, which was associated with increased feelings of anxiety and loneliness and loneliness in turn was related to higher rates of depression. These findings show that HRGC individuals appear to be more worried about their own health during the pandemic than LRGC individuals. We assume that HRGC individuals have avoided social contacts to protect themselves from Covid-19 infections. This increase in social isolation may have resulted in the observed higher rates of loneliness in HRCG individuals, which were associated with depressive symptoms. This is in line with a study by Mayerl et al. [36] showing that COVID-19-related social restrictions were associated with feelings of loneliness and predicted depressive symptoms 10 months later. Quadt et al. [37], proposed a model that perceived loneliness may initiate a cascade of complex body-brain interactions responsible for severe mental and physical health problems.

The results clearly show that the mental health burden is higher among persons at increased risk of severe illness from Covid-19 compared to persons at low risk of severe illness from Covid-19. HRGC individuals are more worried about their own health and report more loneliness, anxiety and depressive symptoms. One factor that may counteract feelings of loneliness and low social connectedness is social support. Therefore, social support during lockdown periods is of utmost importance for individuals prone to mental health problems. Consequently, people at increased risk of severe illness from Covid-19 should not only be protected from a Covid infection but should also receive psychosocial support to decrease feelings of loneliness and increase feelings of social connectedness (e.g. chat-based hotlines, online communication platforms) in order to minimize negative consequences for their mental health during periods of lockdown. This is also in line with a recent study showing that greater social connectedness is associated with reduced stress and fatigue during Covid-19 related lockdown [38]. These findings underline the importance of maintaining social connections also during Covid-19 restrictions to reduce depressive symptoms in pandemic situations.

This study has several limitations. Firstly, it needs to be pointed out that cross-sectional data were collected via an online survey tool, which was mainly promoted by German social service organisations. This recruitment process is likely to have caused a selection bias within the sample by primarily reaching individuals in need for advice from those organisations. Hence, the data collected is not representative of the German general population. Accordingly, representative cross-sectional samples and longitudinal data are desirable in future research. Secondly, the outcome instruments used in the survey were not entirely adapted to the time period of interest, i. e. the first lockdown in Germany. Therefore, it remains unclear whether the mental health burdens reported here changed due to the lockdown. Third, we have not measured social withdrawal directly, but only assume that concerns about own health resulted in reduced social contacts, which may explain the association with perceived loneliness.

## Conclusions

This study demonstrates that the mental health burden of the Covid-19 pandemic is high. This is especially true for individuals who are at increased risk of severe illness from Covid-19. These individuals have a particular need for psychosocial support during times of lockdown. Therefore, they should be specifically supported by corresponding offers (e.g. by phone, in chats or online). Moreover, government officials should take into account the mental health consequences of measures aiming at social distancing. More research, however, is needed on possible long-term consequences of social distancing on mental health.

## Data Availability

The datasets generated and/or analyzed during the current study are not publicly available due to reasons of sensitivity but are available from the corresponding author Daniel Deimel on reasonable request.

## Abbreviations

COVID-19: Coronavirus SARS-COV2 disease 2019
SARS-CoV-2: Severe acute respiratory syndrome coronavirus 2
WHO: World Health Organization
RKI: Robert Koch Institut
HRGC: Individuals at increased risk of severe illness from Covid-19
LRGC: Individuals at low risk of severe illness from Covid-19
PHQ: Patient Health Questionnaire
GAD: Generalized Anxiety Disorder
SBQ-R: Suicide Behaviours Questionnaire – Revised
OSSS-3: Oslo Social Support Scale – 3
